# *Rouxiella badensis*, a new bacterial pathogen of onion causing bulb rot

**DOI:** 10.3389/fmicb.2022.1054813

**Published:** 2022-11-30

**Authors:** Mei Zhao, Chris Tyson, Ron Gitaitis, Brian Kvitko, Bhabesh Dutta

**Affiliations:** ^1^Department of Plant Pathology, College of Plant Protection, China Agricultural University, Beijing, China; ^2^Department of Plant Pathology, University of Georgia, Tifton, GA, United States; ^3^Vidalia Onion and Vegetable Research Center, University of Georgia, Lyons, GA, United States; ^4^Department of Plant Pathology, University of Georgia, Athens, GA, United States

**Keywords:** population, ANI, dDDH, lipopeptide, antiSMASH, *alt*, *Rouxiella badensis*

## Abstract

Onion bulb rot can be caused by multiple plant pathogens including bacterial pathogens. During our routine survey of commercial onion farms in 2014, 2020, and 2021, seven putative *Rouxiella* spp. strains were isolated from symptomatic onion samples in Georgia, United States. Upon fulfilling Koch’s postulates on onion, a genome analysis was conducted. Whole-genome indices (ANI and dDDH) showed that the strains belonged to *Rouxiella badensis*. Although the seven *R. badensis* strains were not pathogenic on onion foliage, the strains were able to cause bulb rot and could also produce necrotic lesions in a red onion scale assay. *R. badensis* populations increased significantly and to a level comparable to *P. ananatis* PNA 97-1R in a red onion scale infection assay. The core-genome analysis grouped all onion *R. badensis* strains from Georgia together, and distinct from *R. badensis* strains isolated from other sources and locations. Based on the genome analysis of strains (from the current study and available genomes in the repository), type I, III (Ssa-Esc and Inv-Mxi-Spa types), and V secretion systems are present in *R. badensis* genomes, while type II, IV, and VI secretion systems are absent. However, various secondary metabolite gene clusters were identified from *R. badensis* genomes, and a thiol/redox-associated enzyme gene cluster similar to the *Pantoea alt* cluster mediating thiosulfinate tolerance was also present in onion strains of *R. badensis*. This is the first report of *R. badensis* as a plant pathogen.

## Introduction

The emergence of plant pathogens in agricultural ecosystems represent threats to food security. Onions (*Allium cepa* L.) are one of the most important and widely consumed vegetables in the world (Griffiths et al., 2002). In Georgia, United States, onions are the most valuable vegetable commodity, with an average farm gate value of $145 million (USDA, 2020). Georgia is the largest producer of fresh market spring season onions in the United States, accounting for nearly 63% of the total production (USDA, 2020).

Bacterial pathogens can affect the onion crop at many stages of the crop growth, including seedling stage, mature plant, mature bulb, and bulbs in storage. Pre-and post-harvest bacterial bulb rots pose a challenge to onion growers and most of the production costs related to disease management are utilized to mitigate it (Mark et al., 2002). Numerous bacterial genera and species have been associated with onion bulb rot but only a handful of them have been characterized (Mark et al., 2002). Among the bulb rotting bacteria, members of the family Enterobacteriaceae and Erwiniaceae are common (Mark et al., 2002).

Bacterial pathogens infecting onion utilize diverse mechanisms for pathogenicity and virulence including secretion systems (type II, III, and VI as major plant pathogenicity factors), quorum sensing, and secondary metabolites. Bacterial pathogens infecting onion utilize either one or the combination of these virulence factors. Among the onion-infecting bacterial pathogens, pathogenicity and virulence factors are well characterized in *Pantoea ananatis* (causal agent of center rot of onion) (Asselin et al., 2018; Stice et al., 2020; Polidore et al., 2021). This bacterium can cause both foliar and bulb symptoms and utilize a diverse set of virulence factors during infection in these tissue types (Asselin et al., 2018, Stice et al., 2020, Polidore et al., 2021). Asselin et al. (2018) identified a novel chromosomal gene cluster (HiVir) that is present in onion pathogenic *P. ananatis* strains, but absent in onion non-pathogenic strains. Further, Polidore et al. (2021) confirmed that the HiVir gene cluster in *P. ananatis* encodes the biosynthetic pathway for a small molecular weight toxin termed pantaphos (2-(hydroxy[phosphono]methyl) maleate) that was sufficient to cause rot in onion bulbs. Additionally, the *alt* (allicin tolerance) cluster is important for the colonization of *P. ananatis* in necrotic onion tissue (Stice et al., 2020). The gene cluster also confers tolerance to endogenous thiosulfinates, a class of reactive sulfur antimicrobials found in *Allium* plants after tissue damage, allowing *P. ananatis* to proliferate in onion bulb tissue.

In this manuscript, we report a new bacterial pathogen of onion from the family Yersiniaceae (*Rouxiella badensis*), which is capable of causing onion bulb rot. We also provide a detailed genomic and pathological characterization of this pathogen. Further, we sought to evaluate virulence factors in onion-pathogenic *R. badensis* and assess genomic features/genes potentially conferring pathogenicity and virulence in these onion-pathogenic strains.

## Materials and methods

### Bacterial isolation and inoculum preparation

Symptomatic onion samples including leaves and bulbs were collected from two counties (Tattnall County and Toombs County) in the Georgia Department of Agriculture approved Vidalia-onion growing zone in Georgia, United States, in 2014, 2020, and 2021. Most of the bacterial pathogens identified during this survey belonged to the genera *Pantoea*, *Pseudomonas*, and *Burkholderia*. The source, year of isolation, and county of origin in Georgia for these strains are shown in Table 1.

**Table 1:** List of *Rouxiella badensis* strains, source and location of isolation, and associated characteristics.

Strain	Year	Source of isolation	Location	Onion foliar pathogenicity^a^	Red-scale necrosis and bulb pathogenicity^b^
Rb14-3	2014	Onion	Unknown location in GA	Negative	Positive
20GA0206	2020	Leaf	Toombs County, GA	Negative	Positive
20GA0316	2020	Bulb	Tattnall County, GA	Negative	Positive
20GA0366	2020	Bulb	Tattnall County, GA	Negative	Positive
20GA0383	2020	Bulb	Toombs County, GA	Negative	Positive
20GA0386	2020	Bulb	Toombs County, GA	Negative	Positive
21GA0654	2021	Leaf	Tattnall County, GA	Negative	Positive

a Strain ability to cause necrosis on onion foliage at 5 days post-inoculation (dpi).

b Strain ability to cause necrosis on the red onion scale at 4 dpi and rot of onion bulb at 10 dpi.

Onion bulb symptoms were observed in commercial onion operations, with less than 1% of the bulbs displaying symptoms. Symptoms on onion bulbs included discoloration of internal scales (Supplementary Figure S1). For bacterial isolation, the margins of healthy and symptomatic onion bulb/leaf tissues were excised using a sterile scalpel and macerated in sterile distilled water (sdH_2_O). The tissue macerates were streaked onto nutrient agar (NA) medium. The NA plates were incubated at 28°C for 2 days. Dominant colonies on the isolation plates were selected and streaked onto NA plates to obtain pure cultures.

*Rouxiella badensis* strains were routinely cultured on NA at 28°C for 2 days. For positive experimental control, we used *P. ananatis* strain PNA 97-1R, which is a well-characterized onion foliar and bulb pathogenic strain. When required, media were supplemented with rifampicin at 30 μg/mL. For inoculum preparation, strains were cultured in nutrient broth at 28°C in a rotary shaker (MaxQ 4,450, Thermo Fisher Scientific, Waltham, MA, United States) at 200 rpm for approximately 16 h. Subsequently, the cultures were centrifuged at 16,100 × *g* for 1 min and the supernatants were decanted. The resulting pellets were resuspended in sdH_2_O. The bacterial concentrations were then adjusted to an optical density of 0.3 at 600 nm [~ 10^8^ colony forming units (CFU/mL)] using a Biophotometer (Eppendorf, Hamburg, Germany).

### Pathogen identification

#### 16S rRNA sequencing

The 16S rRNA gene of the seven onion strains was amplified using primers 27F and 1492R (Frank et al., 2008), and the resulting products were sequenced with primers 27F and 1492R using Sanger sequencing by Eurofins genomics (Louisville, KY). The partial gene sequences of 16S rRNA were deposited in GenBank (accession numbers OL635543 to OL635549). The sequences were subjected to a BLAST search against the National Center for Biotechnology Information (NCBI) database. The type strain 16S rRNA sequences of each genus in the Yersiniaceae family, as well as the outgroup *Hafnia alvei* ATCC 13337^T^ (Family Hafniaceae) were downloaded from the NCBI database. The sequences were aligned using MAFFT (v 7.294b) (Katoh and Standley, 2013) in Geneious Prime (v2019.2.3). The alignment of 16S rRNA gene sequences (1,282 nucleotides) was used to construct a maximum-likelihood phylogenetic tree using the PhyML package (v 3.3.20180621) (Guindon et al., 2010). The model was selected using the Akaike Information Criterion (AIC) statistic within jModelTest v 2.1.10 (Darriba et al., 2012). The robustness of the topology was estimated using 1,000 bootstrap replicates. The tree was visualized using R package ggtree v3.0.2 (Yu et al., 2017).

##### Genome sequencing

For bacterial species determination, the whole-genomes of the seven onion strains were sequenced. For sequencing, cultures for each bacterial strain were prepared by transferring single colonies from two-day-old NA plates into 4 mL of nutrient broth followed by incubation at 28°C overnight in a rotary shaker at 200 rpm. Total microbial genomic DNA was extracted from 1 mL of an overnight culture using a Monarch Genomic DNA Purification Kit (New England Biolabs, Ipswich, MA, United States) according to the manufacturer’s instructions. The genomic libraries were prepared using a NEBNext Ultra II DNA Library Prep Kit for Illumina and were sequenced using an Illumina Novaseq 6,000 platform by Novogene Co., Ltd. (Beijing, China). The raw sequences were filtered using fastp v 0.20.0 http://www.bioinformatics.babraham.ac.uk/projects/fastqc/, and quality-checked using fastqc v 0.11.9. Processed reads were assembled using SPAdes v 3.14 (--isolate --cov-cutoff auto mode) (Bankevich et al., 2012) and filtered to a minimum contig size of 500 bp. The assembly files were deposited in the NCBI database under the BioProject PRJNA776088. The final assemblies were also uploaded to the Life Identification Number (LIN) platform (Tian et al., 2020).

##### Digital DNA–DNA hybridization and average nucleotide identity

The species-level taxonomic position of the seven onion strains was determined by comparing the dDDH and the ANI values for the seven onion strains and the closely related type strains. The dDDH values were calculated using the recommended settings (formula 2) of the genome-to-genome distance calculator 2.1 (Meier-Kolthoff et al., 2013) using the Type Strain Genomic Server (TYGS) (Meier-Kolthoff and Göker, 2019). The pairwise ANI values based on BLAST (ANIb) were calculated using jSpeciesWS v1.2.1 (Richter et al., 2016). The whole-genome sequence of the type strains of *Rouxiella* spp. were used for the analysis and they were as follows: *R. badensis* DSM 100043^T^ (RefSeq assembly accession: GCF_002093665.1), *R. silvae* 213^T^ (RefSeq assembly accession: GCF_002093625.1), *R. chamberiensis* 130333^T^ (RefSeq assembly accession: GCF_000951135.1), and *R. aceris* SAP-1^T^ (RefSeq assembly accession: GCF_012933545.1).

#### Phylogenetic analysis of housekeeping genes *atpD*, *gyrB*, *infB*, and *rpoB*

The gene sequences of *atpD*, *gyrB*, *infB*, and *rpoB* were extracted from genomes of seven *R. badensis* onion strains and selected type strains using BLASTN and analyzed in Geneious Prime (v2019.2.3). The individual gene sequences were aligned using MAFFT (v 7.294b) (Katoh and Standley, 2013). The alignments of each gene sequence (*atpD* 1,383 bp; *gyrB* 2,391 bp; *infB* 2,720 bp; *rpoB* 4,029 bp) were used to construct maximum-likelihood phylogenetic trees using the PhyML package (v 3.3.20180621) (Guindon et al., 2010). The model was selected using the AIC statistic within jModelTest v 2.1.10 (Darriba et al., 2012). The robustness of the topology was estimated using 1,000 bootstrap replicates.

#### Phylogenomic analysis

The seven *R. badensis* (from onion) genomes along with three other *R. badensis* genomes available on GenBank (SER3 (RefSeq assembly accession: GCF_011067265.1), C173 (RefSeq assembly accession: GCF_014859005.1), and DSM 100043^T^ (RefSeq assembly accession: GCF_002093665.1)), and *R. silvae* 213^T^ (as an outgroup) were uploaded to the M1CR0B1AL1Z3R web server^1^ for phylogenetic analyses based on core genomes (Avram et al., 2019). The settings (maximal e-value cutoff: 0.01, identity minimal percent cutoff: 70.0%, minimal percentage for core: 100.0%) were used with bootstrap analyses enabled. Similarly, in addition to the 10 *R. badensis* genomes, 10 genomes of *Rouxiella* spp. and the type strains of the type species in each genus in Yersiniaceae family were included in phylogenomic analysis using the M1CR0B1AL1Z3R web server. *Hafnia alvei* ATCC13337^T^ was used as an outgroup. The settings (maximal e-value cutoff: 0.01, identity minimal percent cutoff: 70.0%, minimal percentage for core: 50.0%) were used with bootstrap analyses enabled.

### Pathogenicity on onion foliage, bulb, and on red-onion scale

Foliar inoculation assays were conducted as described by Koirala et al. (2021). Briefly, onion (cv. Century) seedlings were established in plastic pots containing commercial potting mix and maintained in a greenhouse at ~25°C. Eight-week-old onion seedlings were inoculated after cutting the leaf 1 cm from the apex with a pair of scissors sterilized with 70% ethanol. Using a micropipette, 10 μL of 1 × 10^8^ CFU/mL bacterial suspension (~ 1 × 10^6^ CFU/leaf) was placed at the cut end of the leaf twice, diagonally opposite to each other. Seedlings inoculated with *P. ananatis* PNA 97-1R and sdH_2_O as described above were used as positive and negative controls, respectively. At 5 days post-inoculation (dpi), inoculated onion seedlings were evaluated for symptom development. Three seedlings per strain were used for one experiment and the experiment was conducted twice.

Whole bulb inoculation assays were conducted as previously described (Koirala et al., 2021). Briefly, after removal of outer dry tunicate layers, onion bulbs (cv. Century) were surface sterilized with 70% ethanol, and inoculated at the shoulder with 500 μL of a 1 × 10^8^ CFU/mL bacterial suspension using a syringe and a sterile needle. Bulbs were incubated on an aluminum tray at room temperature. At 10 dpi, bulbs were cut open vertically through the inoculation point and photographed. Bulbs inoculated with *P. ananatis* PNA 97-1R and sdH_2_O as described above were used as positive and negative controls, respectively. Strains that caused internal bulb rot were considered pathogenic. The inoculated bacteria were re-isolated from the symptomatic onion bulb tissues and the identities of the isolated bacteria were confirmed using 16S rRNA gene based sequencing. Three bulbs per strain were used for one experiment and the experiment was conducted twice.

For red onion scale inoculation, *R. badensis* strains were inoculated on red onion scale as previously described (Stice et al., 2018). Briefly, red onion bulbs (cv. Red Barret) were sliced into small squares (approximately 2 cm × 2 cm), sterilized in 0.5% sodium hypochlorite, and washed with tap water. A 10 μL pipette tip was used to penetrate the onion scale at the center with finger pressure. Ten microliters of a 1 × 10^8^ CFU/mL bacterial suspension (1 × 10^6^ CFU) were deposited at the wounded area. The sdH_2_O was used as the negative control and *P. ananatis* PNA 97-1R was used as the positive control. The onion scales were incubated at room temperature, and photographed at 4 dpi. Strains that caused scale necrosis were considered pathogenic. The inoculated bacteria were re-isolated and confirmed from the symptomatic inoculated onion scales as described above. Three replicates per strain were used for one experiment and the experiment was conducted twice.

To further ascertain that *R. badensis* is pathogenic on onion, we assessed population dynamics of *R. badensis* on red onion scales. For this assay, a spontaneous rifampicin-resistant *R. badensis* strain (20GA0316) was selected. The strain 20GA0316R was inoculated onto the red onion scale as described above. *Pantoea ananatis* PNA 97-1R (rifampicin-resistant) and PANS 99-23R (rifampicin-resistant) were used as positive and negative control, respectively. These strains were well-characterized previously (Stice et al., 2018). The strain (PNA 97-1R) is pathogenic on onion whereas strain PANS 99-23R is non-pathogenic (Stice et al., 2018). At 1, 2, and 3 dpi, scale tissue samples (approximately 0.2 cm × 0.2 cm) were cut using a sterile blade 0.5 cm away from the inoculation point, weighed, and placed in 2 mL tubes containing three sterile glass beads (3 mm) and 500 μL sdH_2_O. Tissue samples were macerated in a bead mill homogenizer (Omni International Inc., Kennesaw, GA) four times for 30 s each at 4 m/s speed. A 10-fold dilution series of the macerates was made in sdH_2_O to 10^−6^ and each dilution was plated as 10 μL droplets on Lysogeny Broth (LB) agar amended with rifampicin. Colonies were counted 2 days after incubation and converted to Log_10_ CFU/g. Six replicates per strain were used for one experiment and the experiment was conducted twice. Analysis of variance was used to analyze the three treatments at each time point, and significant differences (*p* = 0.05) among treatments were determined according to Tukey–Kramer’s honestly significant difference test.

### Biochemical tests

Carbon source utilization and tests for sensitivity to different chemicals were determined using the BIOLOG GEN III MicroPlate system (BIOLOG, Hayward, CA). Bacterial cells from nutrient agar were suspended in BIOLOG Inoculation Fluid and adjusted to 95% transmission (optical density = 0.02). Further, 100 μL of the suspension were added to each well of the GENIII MicroPlate and incubated for 48 h at 28°C. The results were recorded manually.

### Genome enabled identification of virulence factors and secondary metabolite gene clusters

Genome annotation was performed using Rapid Annotation using Subsystem Technology (RAST v2.0) server (Overbeek et al., 2014). Annotated genomes were assessed manually in the RAST server and in Geneious Prime (v 2019.2.3) and searched using BLAST for the presence of common virulence elements including typical protein secretion systems. EffectiveDB^2^ was also used to predict the presence of type III, IV, and VI secretion systems (Eichinger et al., 2016). In addition, protein secretion systems (types I, II, III, IV, V, and VI) were also predicted using TXSScan/MacSyFinder (Abby et al., 2014, 2016). To characterize the type of T3SSs present in *R. badensis,* the amino acid sequences of the major export apparatus protein SctV from representative T3SS types were downloaded from NCBI. The amino acid sequences were aligned using MAFFT (v 7.294b) (Katoh and Standley, 2013) and the alignment was used to generate a neighbor-joining tree using the Jukes-Cantor model (Jukes and Cantor, 1969) within Geneious Prime 2019.2.3. The bootstrap support values were calculated using 1,000 replicates. The assembly files were used as input data for *in silico* secondary metabolite gene cluster analysis using antiSMASH v 6 with default parameters and the ‘knownclusterblast’ flag (Blin et al., 2021).

## Results

### Pathogen identification

**Figure 1:**
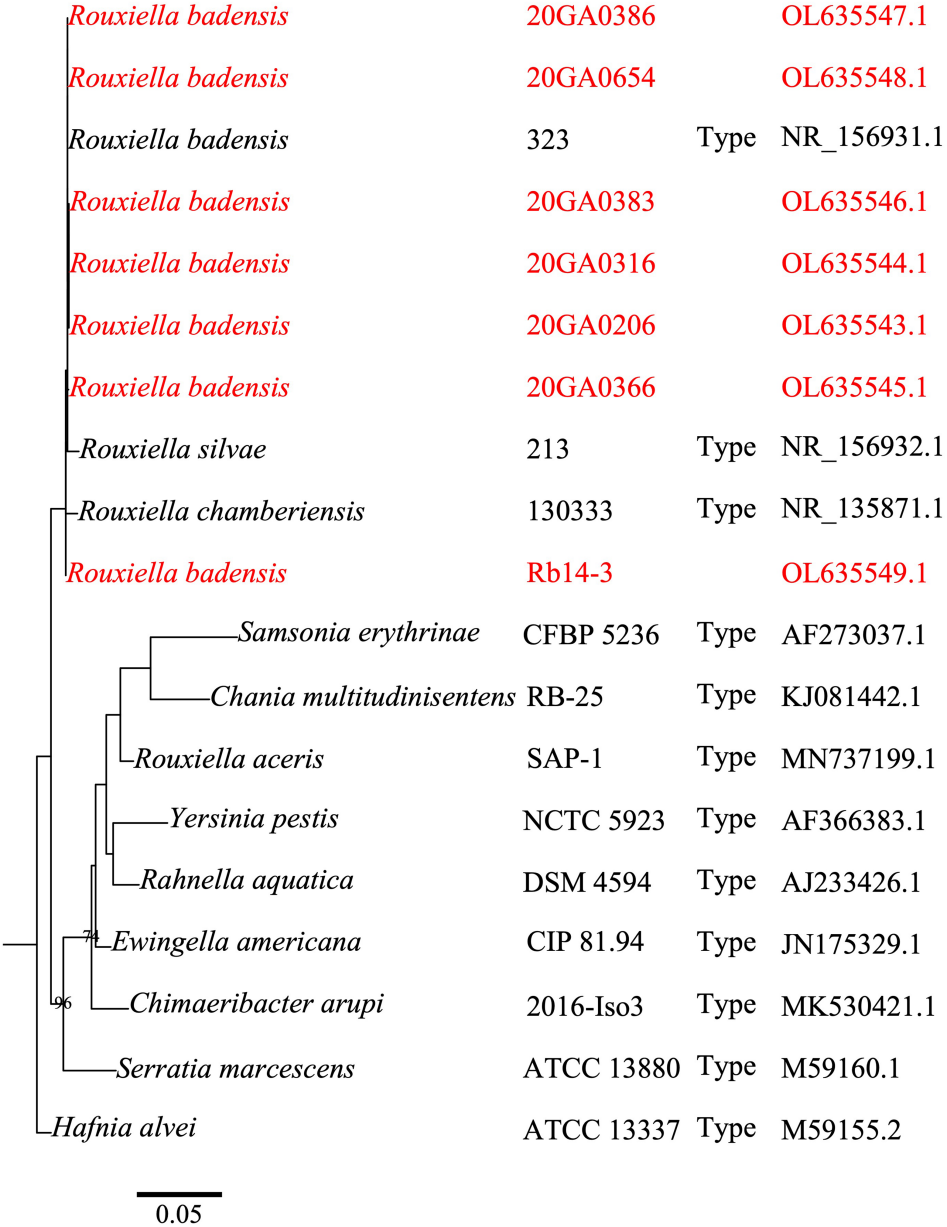
A phylogenetic tree based on partial gene 16S rRNA sequences of *Rouxiella badensis* strains from onion and closely related species in the Yersiniaceae family. *Hafnia alvei* ATCC 13337^T^ (Family Hafniaceae) was used as the outgroup. The 16S rRNA gene sequences (1,282 nucleotides) were aligned using MAFFT. The alignment was used to construct a maximum-likelihood tree using the PhyML package. The model was selected using the Akaike Information Criterion statistic within jModelTest v 2.1.10. Bootstrap values (>70%) from 1,000 replicates are shown at the nodes. The bar indicates number of nucleotide changes per site. The species name, strain name, type strain status, and accession numbers are shown. The tree was visualized using R package ggtree. Strains in red are *R. badensis* strains isolated from onion in Georgia.

The partial 16S rRNA sequences of seven onion strains showed 99.9 to 100.0% identity with *R. badensis* strain 323^T^ (GenBank accession: KX784906.1). The 16S rRNA phylogenic tree showed that the seven onion strains clustered together with the type strains of *R. badensis*, *R. silvae,* and *R. chamberiensis* (Figure 1). The ANI and dDDH values between seven onion strains and type strains of *Rouxiella* members were estimated. The dDDH values ranged from 97.7 to 98.0% compared to *R. badensis* DSM 100043^T^, and were less than 24.0% when compared to *R. silvae* 213^T^, *R. chamberiensis* 130333^T^, and *R. aceris* SAP-1^T^ (Table 2). The ANIb values ranged from 99.4 to 99.7% compared to *R. badensis* DSM 100043^T^, and were less than 80.4% when compared to *R. silvae* 213^T^, *R. chamberiensis* 130333^T^, and *R. aceris* SAP-1^T^ (Table 2). The LINs for seven *R. badensis* onion strains are shown in Table 3. Interestingly, the LIN platform reported that *R. badensis* strain 20GA0386 was 99.9% similar to *R. badensis* strain 20GA0383.

**Table 2:** Genomic relationship between *Rouxiella badensis* strains and the type strains of *Rouxiella* species.

Strain	*R. badensis* DSM 100043^T^		*R. silvae* 213^T^		*R. chamberiensis* 130333^T^		*R. aceris* SAP-1^T^	
	dDDH (%)	ANIb (%)	dDDH (%)	ANIb (%)	dDDH (%)	ANIb (%)	dDDH (%)	ANIb (%)
Rb14-3	97.8	99.5	23.9	80.3	23.7	79.9	22.7	78.2
20GA0206	98.0	99.4	23.9	80.4	23.8	79.9	22.7	78.2
20GA0316	98.0	99.6	24.0	80.3	23.7	79.9	22.7	78.1
20GA0366	97.8	99.6	24.0	80.3	23.7	79.8	22.7	78.2
20GA0383	98.0	99.7	24.0	80.4	23.7	79.8	22.7	78.2
20GA0386	98.0	99.7	24.0	80.4	23.7	79.8	22.7	78.2
21GA0654	98.1	99.6	24.0	80.3	23.7	79.9	22.7	78.1

Average nucleotide identity (ANI) and digital DNA–DNA hybridization (dDDH) values were calculated using JSpeciesWS v1.2.1 and Genome-to-Genome Distance Calculator 2.1 (formula 2), respectively.

**Table 3:** Life identification number of *Rouxiella badensis* onion strains and type strain.

Strain	A	B	C	D	E	F	G	H	I	J	K	L	M	N	O	P	Q	R	S	T
DSM 100043^T^	51	11	0	3	0	0	0	0	0	0	0	0	0	0	0	0	0	0	0	0
20GA0206	51	11	0	3	0	0	0	0	0	0	0	0	0	1	0	0	0	0	0	0
20GA0316	51	11	0	3	0	0	0	0	0	0	0	0	0	1	1	0	0	0	0	0
20GA0366	51	11	0	3	0	0	0	0	0	0	0	0	0	2	0	0	0	0	0	0
20GA0383	51	11	0	3	0	0	0	0	0	0	0	0	0	3	0	0	0	0	0	0
20GA0386	51	11	0	3	0	0	0	0	0	0	0	0	0	3	0	0	0	0	0	0
20GA0654	51	11	0	3	0	0	0	0	0	0	0	0	0	1	1	0	0	0	0	1
Rb14-3	51	11	0	3	0	0	0	0	0	0	0	0	0	4	0	0	0	0	0	0

**Figure 2:**
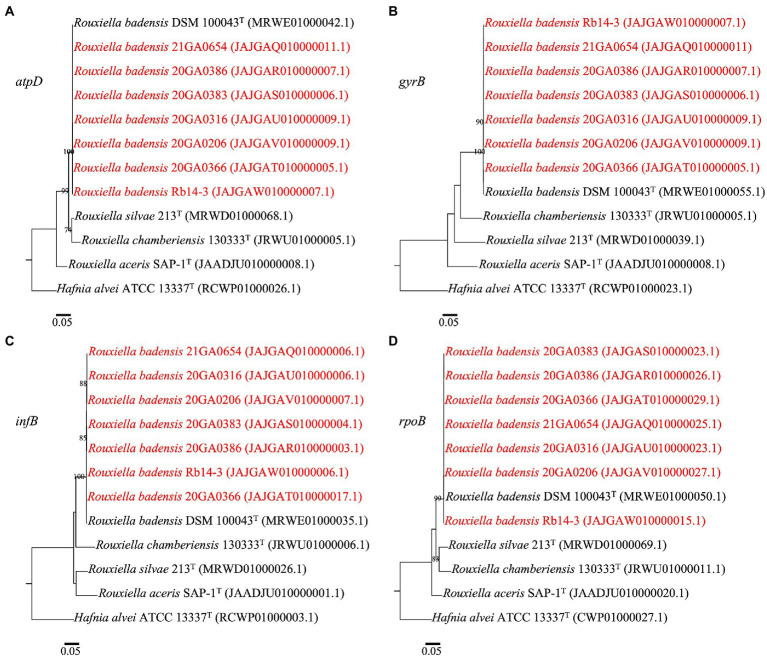
Phylogenetic trees based on sequences of *atpD* **(A)**, *gyrB* **(B)**, *infB* **(C)**, and *rpoB* **(D)** of *Rouxiella badensis* strains from onion and existing *Rouxiella* species. *Hafnia alvei* ATCC 13337^T^ was used as the outgroup. The individual housekeeping genes were aligned using MAFFT. The alignment was used to construct a maximum-likelihood tree using the PhyML package. The model was selected using the Akaike Information Criterion statistic within jModelTest v 2.1.10. Bootstrap values (>70%) from 1,000 replicates are shown at the nodes. The bar indicates number of nucleotide changes per site. Strains in red are *R. badensis* strains isolated from onion in Georgia.

In the phylogenetic trees for each gene (*atpD*, *gyrB*, *infB*, and *rpoB*), all seven *Rouxiella* strains formed a distinct clade with *R. badensis* DSM 10043^T^ with 99 to 100% bootstrap values (Figure 2). The 1,382 bp out of 1,383 bp (99.9%) *atpD* sequence were identical among *R. badensis* onion strains and the type strain. The 2,384 bp out of 2,391 bp (99.7%) *gyrB* sequence were identical among *R. badensis* onion strains and the type strain. The 2,683 bp out of 2,696 bp (99.5%) *infB* sequence were identical among *R. badensis* onion strains and the type strain. The 4,021 bp out of 4,029 bp (99.8%) *rpoB* sequence were identical among *R. badensis* onion strains and the type strain.

**Figure 3:**
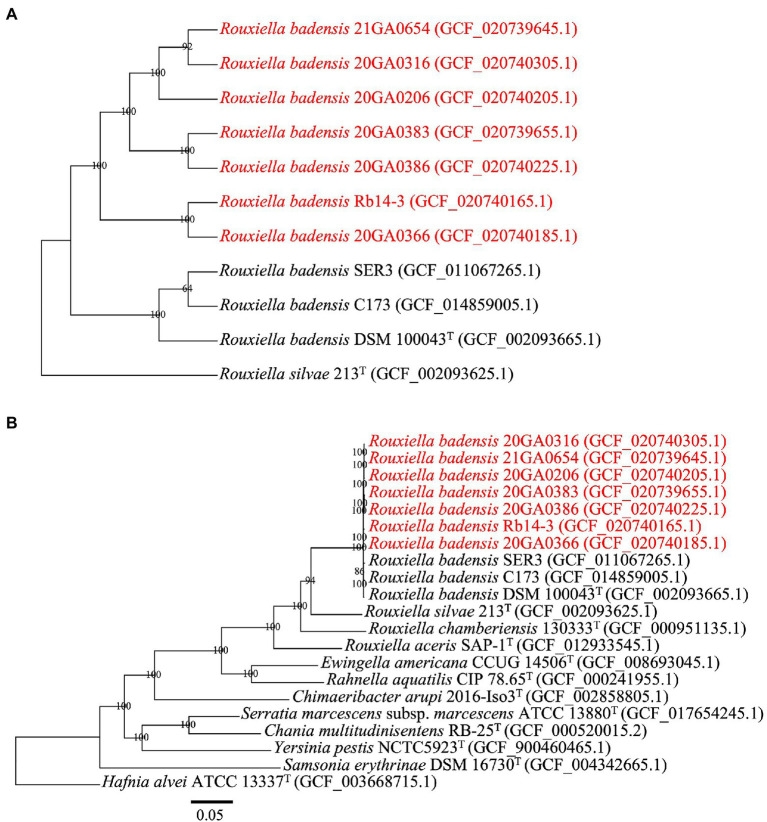
Phylogenomic analysis of *Rouxiella badensis* strains based on whole-genome-sequences. The tree was generated using the M1CR0B1AL1Z3R web server. The bootstrap support values are shown at the node. Strains in red are *R. badensis* strains isolated from onion in Georgia. **(A)** A phylogenetic tree based on the concatenated amino acid sequences (1,123,094 amino acid positions) of 3,393 core genes defined in the 11 *Rouxiella* genomes by the M1CR0B1AL1Z3R web server. *Rouxiella silvae* 213^T^ was used as the outgroup. **(B)** A phylogenetic tree based on the concatenated amino acid sequences (1,195,559 amino acid positions) of 3,532 core genes defined in the 21 genomes by the M1CR0B1AL1Z3R web server. *Hafnia alvei* ATCC 13337^T^ was used as the outgroup. The bar in **(B)** indicates number of amino acid changes per site.

The core-proteome (3,393 genes) alignment of 10 *R. badensis* strains with *R. silvae* 213^T^ as an outgroup performed at the M1CR0B1AL1Z3R web server is shown in Figure 3A. The topology of the maximum-likelihood-based core-proteome tree showed the seven onion *R. badensis* strains formed one major clade while the non-onion *R. badensis* strains (strains SER3, C173, and DSM 100043^T^) formed a different clade with 100% support values (Figure 3A). Among all seven onion *R. badensis* strains, a total of 12,104 amino acid differences out of the 1,123,094 amino acid core-protein-alignment were found. Regarding the pairwise comparison between *R. badensis* strains (21GA0654 vs. 20GA0316) within the same cluster contained 5,827 amino acid differences out of the 1,123,094 amino acid aligned. Strain 20GA0383 and 20GA0386 within the same cluster possessed 6,323 amino acid differences. Strain Rb14-3 and 20GA0366 within the same cluster contained a difference of 8,321 amino acids. In addition, the core-proteome (3,532 genes) alignment of 20 strains in Yersiniaceae family including 10 *R. badensis* strains with *H. alvei* ATCC 13337^T^ as an outgroup is shown in Figure 3B. The phylogenomic tree of 21 strains showed that all 10 *R. badensis* strains clustered together, with *R. silvae* 213^T^ as the closest neighbor (Figure 3B).

### Pathogenicity on onion foliage, bulb, and on red-onion scale

The pathogenicity of each strain on onion was assessed using foliar and bulb assays, and a red-onion scale necrosis assay. The *R. badensis* onion strains (*n* = 7) and sdH_2_O control did not cause any symptoms on the onion leaf while the positive control *P. ananatis* PNA 97-1R induced typical water-soaking and foliar necrotic symptoms at 5 dpi (data not shown).

**Figure 4:**
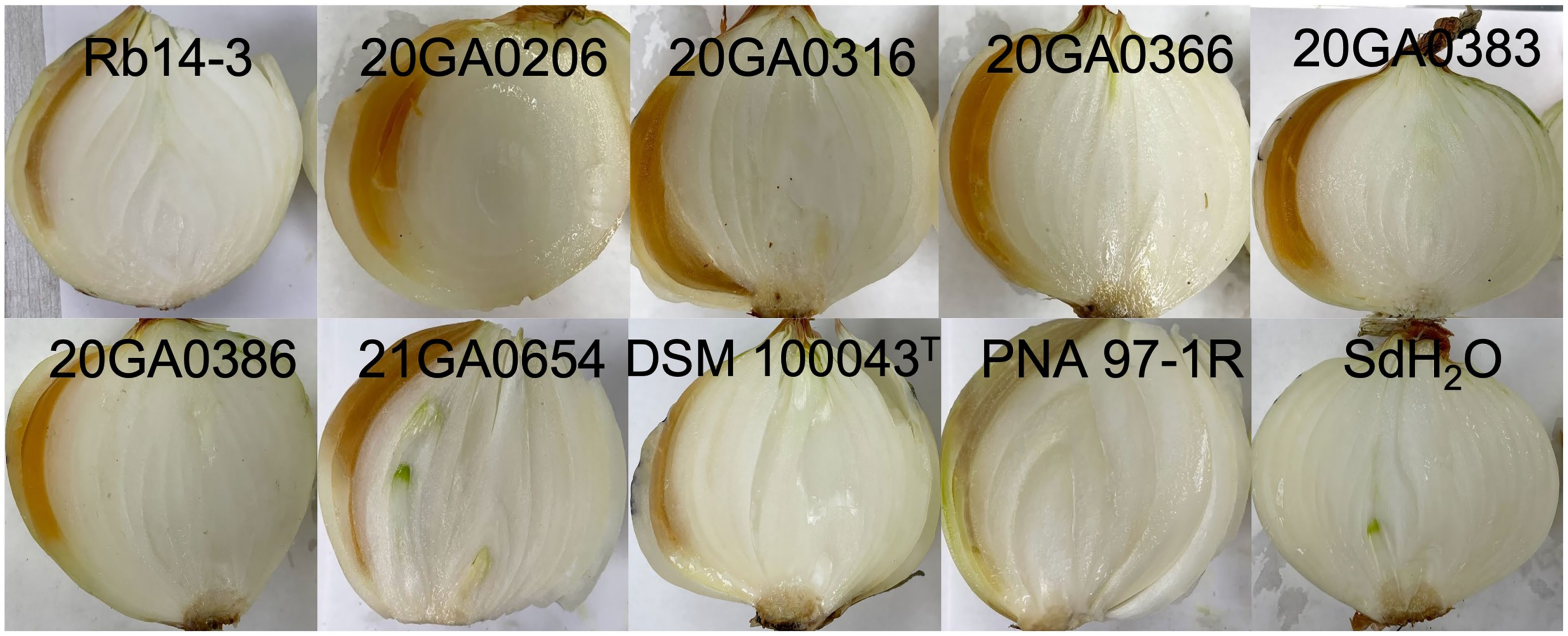
Pathogenicity of *Rouxiella badensis* strains on whole onion bulbs. Onion bulbs (cv. Century) were purchased from the store, surface disinfected with 70% ethanol, and inoculated with 1  ml of cell suspensions containing 10^8^  CFU/mL at the shoulder using a syringe attached with a needle. *Pantoea ananatis* PNA 97-1R and sterilized distilled water (sdH_2_O) were used as positive and negative controls, respectively. Onion bulbs were cut open and photographed at 10  days post-inoculation.

The *R. badensis* onion strains (*n* = 7) induced bulb symptoms causing orange tissue discoloration at 10 dpi. While the positive control bulbs inoculated with *P. ananatis* strain PNA 97-1R developed brown discolored tissues, the negative control bulbs inoculated with sdH_2_O did not develop any visible symptoms. Interestingly, *R. badensis* type strain DSM 10043^T^ also induced bulb symptoms (Figure 4). *Rouxiella badensis* were recovered from symptomatic tissues and their identities were tentatively ascertained with 16S rRNA sequences.

**Figure 5:**
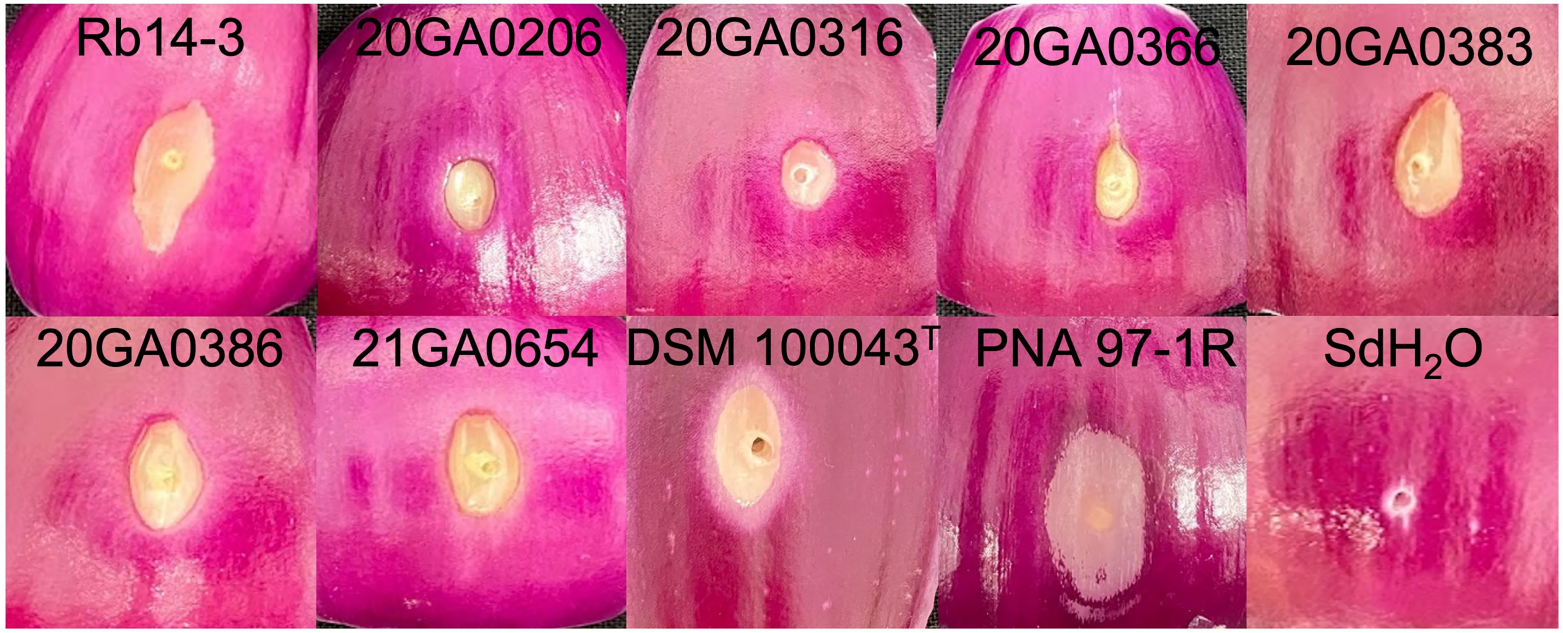
Red-scale necrosis phenotype of *Rouxiella badensis* strains. Red onion bulbs were purchased from the store, sliced into squares, surface disinfected with 10% bleach, wound at the center using a pipette tip, and inoculated with 10 μL of cell suspensions containing 10^8^  CFU/mL. *Pantoea ananatis* PNA 97-1R and sterilized distilled water (sdH_2_O) were used as positive and negative controls, respectively. The symptoms were photographed at 4  days post-inoculation.

**Figure 6:**
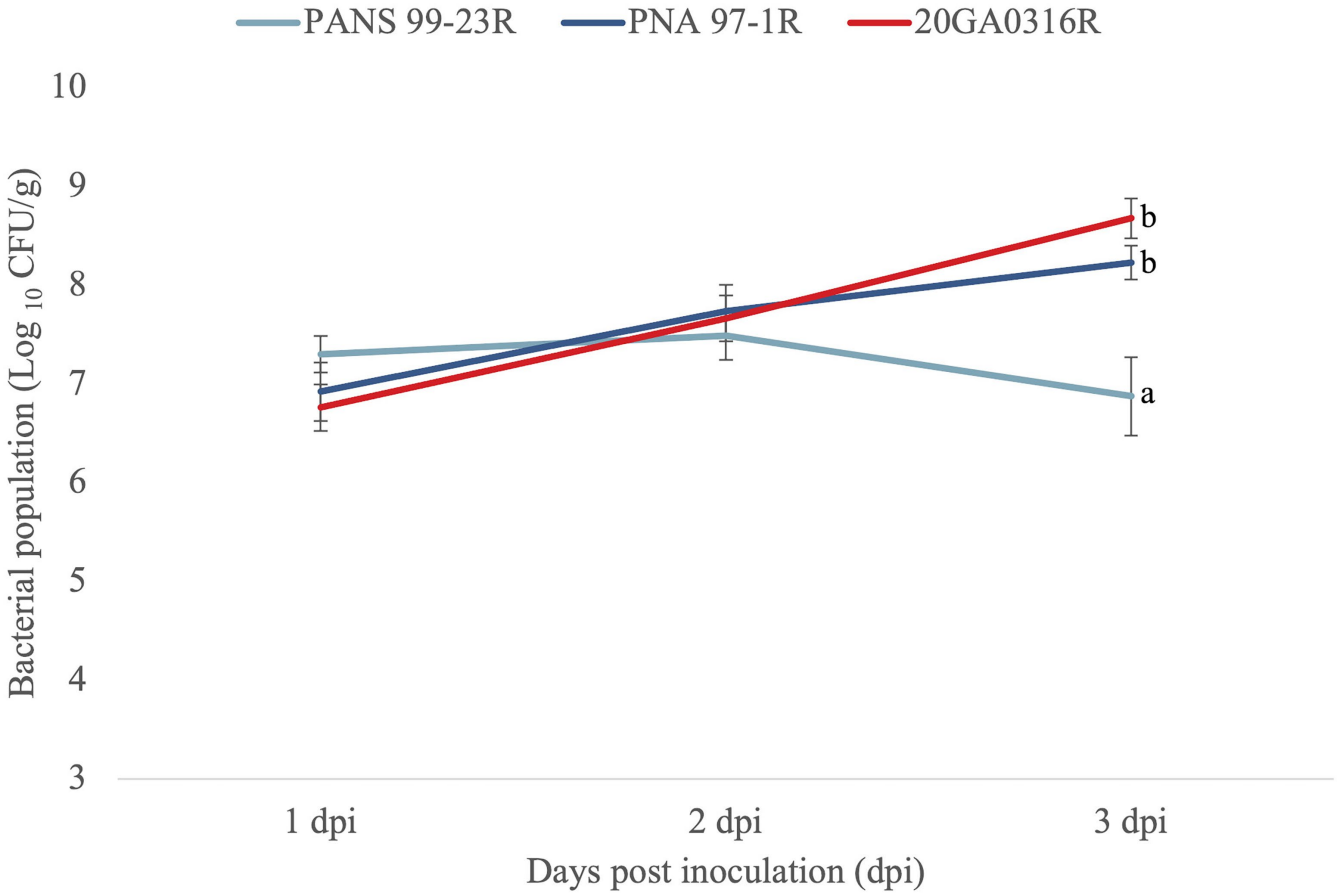
Population dynamics of *Rouxiella badensis* 20GA0316R over time in red onion scale tissues, compared to negative control (a known red-scale necrosis negative) strain *Pantoea ananatis* PANS 99-23R, and positive control *P. ananatis* PNA 97-1R. Strains were inoculated onto red onion scales with 10 μL of cell suspensions containing 10^8^ CFU/mL. At 1, 2, and 3 days post-inoculation (dpi), tissue samples (0.2 cm × 0.2 cm) were taken 0.5 cm away from the inoculation point, weighed, and macerated in sterilized distilled H_2_O, and plated on LB agar with rifampicin. Colonies were counted 2 days after incubation and converted to Log_10_ CFU/g. The experiment was conducted twice and each treatment was replicated six times per experiment. The mean population and the standard error of the mean are shown. Different letters indicate significant differences (*p* = 0.05) among treatments according to Tukey–Kramer’s honestly significant difference test.

All seven onion strains were able to cause necrosis on red onion scales. The symptoms caused by *R. badensis* strains started developing as early as 1 dpi near the inoculation point, and were different from the scale clearing symptoms caused by *P. ananatis* PNA 97-1R (Figure 5). *Rouxiella badensis* type strain DSM 10043^T^ also caused necrosis on red onion scales (Figure 5). The populations of the *R. badensis* test strain 20GA0316R and positive control PNA 97-1R on the red onion scale increased over time from 1 dpi to 3 dpi, while the populations of the negative control strain *P. ananatis* PANS 99-23R decreased over time (Figure 6).

### Phenotypic characteristics

The *R. badensis* colonies were white, round, smooth, with entire edges, and 1.0–2.0 mm in diameter after incubation at 28°C for 24 h on NA medium. The complete list of the BIOLOG GENIII assay result is shown in Supplementary Table S1. Interestingly, *R. badensis* strains were positive for most of the reactions, ranging from 88% (84 out of 95 reactions; strains Rb14-3 and 20GA0206) to 94% (90 out of 95 reactions; strain 20GA0316). All eight *R. badensis* strains (seven onion strains and the type strain) were not able to utilize the following substrates: L-Pyroglutamic acid, p-Hydroxy-phenylacetic acid, D-Lactic acid methyl ester, α-Hydroxy-butyric acid, and α-Keto-butyric acid.

### Genome enabled identification of putative virulence factors and secondary metabolite gene clusters

**Figure 7:**
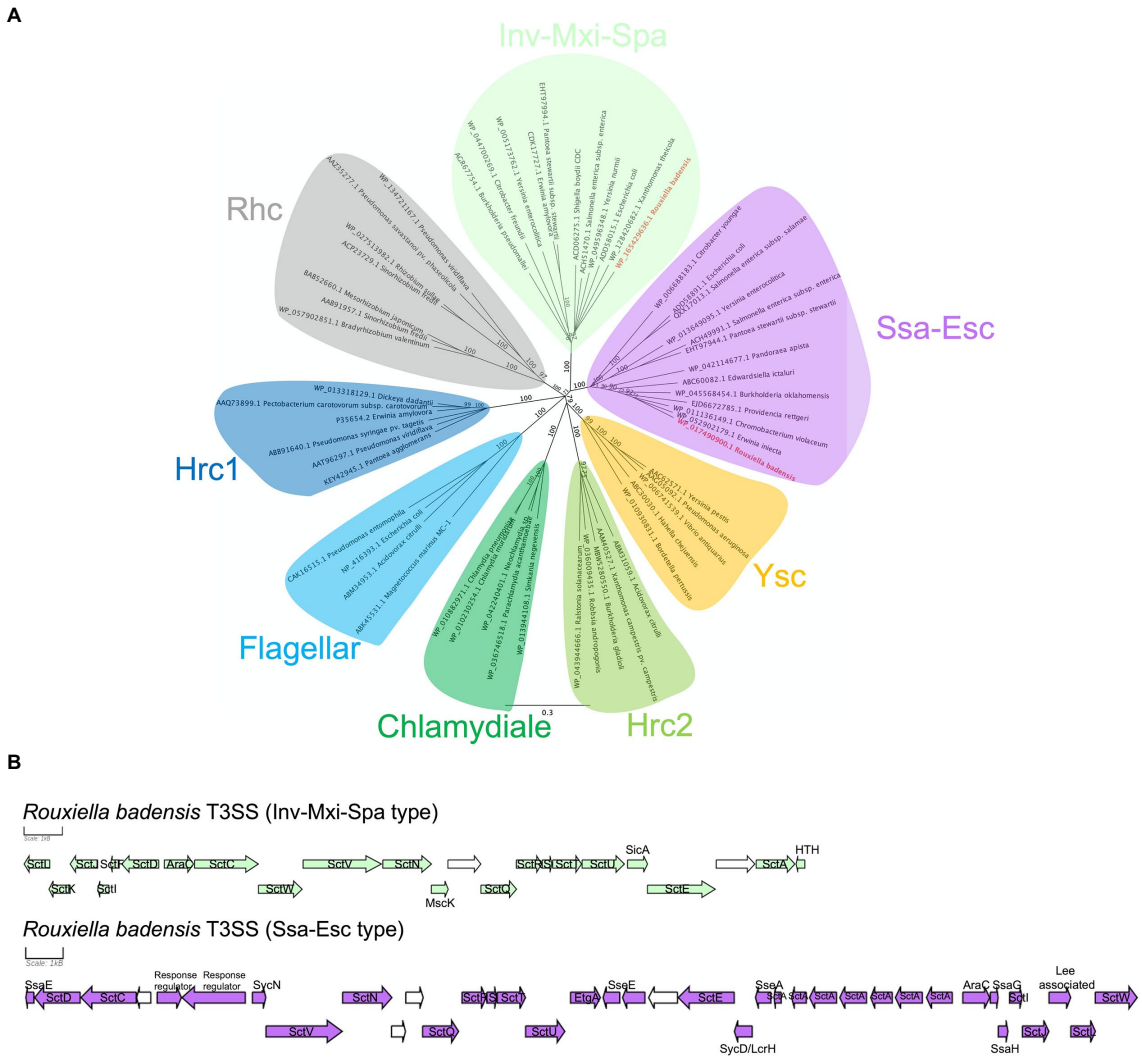
*Rouxiella badensis* type III secretion systems (T3SS). **(A)** Neighbor-joining phylogeny of selected representative T3SSs based on the amino acid sequence of the major export apparatus protein SctV. The amino acid sequences were aligned using MAFFT and the tree was constructed using the Jukes-Cantor method. The bootstrap support values were calculated using 1,000 replicates and shown at the nodes. *Rouxiella badensis* T3SSs are in bold and red. The protein accession numbers are provided at the tip. The bar indicates number of amino acid changes per site. **(B)** *Rouxiella badensis* T3SS gene organizations. The scale bar represents 1 kB. Genes in green and purple have annotations associated with T3SS. Genes in white are predicted to encode hypothetical proteins.

One or two independent sets of type I secretion system (T1SS), one set of type V secretion system (T5SS), and two independent sets of type III secretion system (T3SS) clusters, but no type II, IV, and VI secretion systems were identified in 10 *R. badensis* strains (Table 4). The two T3SS clusters were associated with Ssa-Esc and Inv-Mxi-Spa types of T3SS based on the phylogeny of SctV (Figure 7). Nine secondary metabolite gene clusters in all 10 *R. badensis* genomes identified using antiSMASH were listed in Table 5. Gene clusters associated with a type 1 polyketide synthase (PKS) product, a siderophore (similar to desferrioxamine E), an aryl polyene product, a non-ribosomal peptide synthetase cluster (similar to turnerbactin), a redox-cofactor type product (similar to lankacidin C), a homoserine lactone cluster, a thiopeptide product (similar to O-antigen), a transAT-PKS type lipopeptide, and a T3PKS (similar to venemycin) were identified.

**Table 4:** Protein secretion systems in *Rouxiella badensis* strains based on genome analysis.

Strain	T1SS	T2SS	T3SS	T4SS	T5SS	T6SS
Rb14-3	1*	0	2	0	1	0
20GA0206	1	0	2	0	1	0
20GA0316	1	0	2	0	1	0
20GA0366	1	0	2	0	1	0
20GA0383	2	0	2	0	1	0
20GA0386	2	0	2	0	1	0
21GA0654	1	0	2	0	1	0
SER3	1	0	2	0	1	0
C173	2	0	2	0	1	0
DSM 100043^T^	2	0	2	0	1	0

* Refers to the number of distinct types of each secretion system present in *R. badensis*.

**Table 5:** Secondary metabolite biosynthetic gene clusters in *Rouxiella badensis* strains predicted by antiSMASH v6.

Type	Predicted product	Most similar known cluster
T1PKS^a^	–^e^	–
Siderophore	Other	Desferrioxamine E
Arylpolyene	Other	Aryl polyenes
NRPS^b^	NRP	Turnerbactin
Redox-cofactor	NRP + Polyketide	Lankacidin C
Hserlactone^c^	–	–
Thiopeptide	Saccharide	O-antigen
transAT-PKS, NRPS	NRP: lipopeptide	Tolaasin I / tolaasin F
T3PKS^d^	Polyketide	Venemycin

a T1PKS, type I PKS (polyketide synthase).

b NRPS, non-ribosomal peptide synthetase cluster.

c Hserlactone, homoserine lactone cluster.

d T3PKS, type III PKS.

e –, no information available in the antiSMASH prediction output.

**Figure 8:**
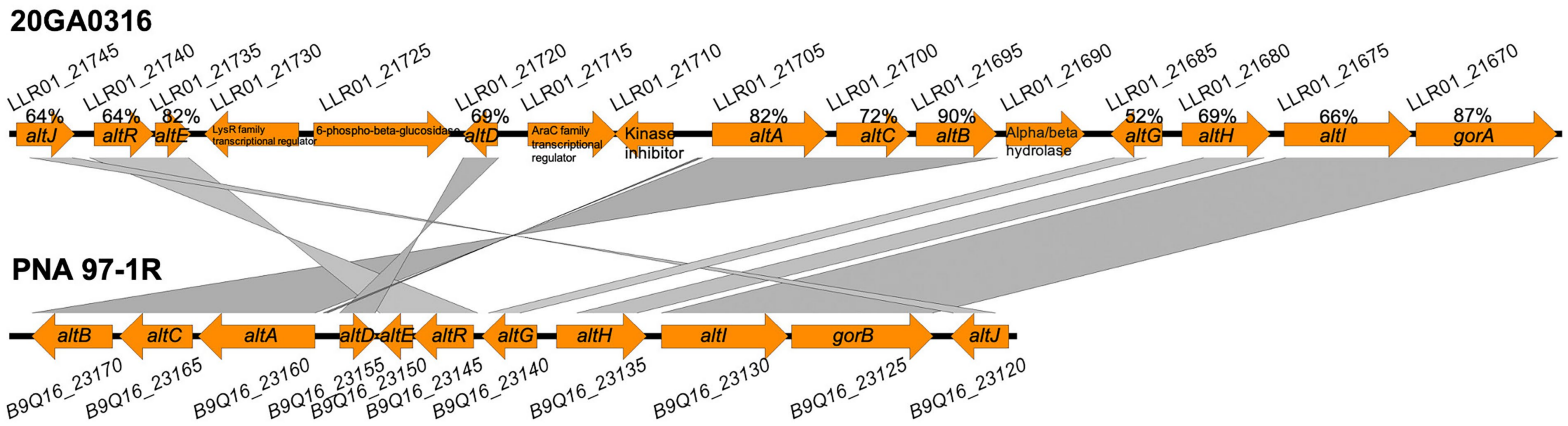
Alignment of *alt* clusters in *Rouxiella badensis* strain 20GA0316 (GenBank accession: GCA_020740305.1) and *Pantoea ananatis* PNA 97-1R (GenBank accession: CP020945), generated with Easyfig program. Gene locus numbers are above and below the gene arrows for 20GA0316 and PNA 97-1R, respectively. The gene annotations are shown inside the orange gene arrow. The protein sequence similarity percentages between homologous proteins are shown above the corresponding gene arrows for 20GA0316.

The HiVir-like gene cluster sequences were not found in any of the 10 *R. badensis* genomes. An *alt-*like gene cluster was found in seven onion-pathogenic *R. badensis* genomes, but not present in *R. badensis* strains that were not associated with onion (Table 6). The *alt-*like gene cluster in the seven onion *R. badensis* has 16 genes, and 11 of them have homology to the *alt* gene cluster in *P. ananatis* (Figure 8). However, those 11 genes between the two *alt* clusters in *R. badensis* and *P. ananatis* are not in synteny (Figure 8). Pairwise protein sequence alignment using BLASTP revealed the similarity percentages of Alt protein sequences range between 52% for AltG to 90% for AltB. The *alt* clusters in *R. badensis* strains from onion include five genes that are absent in the *P. ananatis alt* cluster, including a LysR family transcriptional regulator, a 6-phospho-beta-glucosidase gene, an AraC family transcriptional regulator gene, a kinase inhibitor gene, and an alpha-beta hydrolase gene.

**Table 6:** Potential virulence factors in *Rouxiella badensis* strains available in National Center for Biotechnology Information (NCBI).

Strain	Source	Location of isolation	RefSeq assembly accession	*alt*-like cluster^b^	Lipopeptide^c^
Rb14-3	Onion	Georgia, United States	GCF_020740165.1	Present	Present
20GA0206	Onion	Georgia, United States	GCF_020740205.1	Present	Present
20GA0316	Onion	Georgia, United States	GCF_020740305.1	Present	Present
20GA0366	Onion	Georgia, United States	GCF_020740185.1	Present	Present
20GA0383	Onion	Georgia, United States	GCF_020739655.1	Present	Present
20GA0386	Onion	Georgia, United States	GCF_020740225.1	Present	Present
21GA0654	Onion	Georgia, United States	GCF_020739645.1	Present	Present
SER3	Strawberry fruit	Morelia, Mexico	GCF_011067265.1	Absent	Present
C173	Lowbush blueberries	New Brunswick, Canada	GCF_014859005.1	Absent	Present
DSM 100043^T a^	Peat bog soil	Baden, Germany	GCF_002093665.1	Absent	Present

^a^ T, type strain.

b Presence or absence of the *alt*-like gene cluster based on BLAST analysis.

c Presence or absence of the gene cluster encoding lipopeptide based on antiSMASH analysis.

## Discussion

Onion bulb rot can be associated with various bacterial species. The common causal agents include *Burkholderia* spp., *Pantoea* spp., *Pseudomonas* spp., and *Enterobacter* spp. (Mark et al., 2002). In this study, we report that *Rouxiella* species, particularly *R. badensis* was able to cause onion bulb and scale necrosis. Interestingly, we found *R. badensis* type strain DSM 100043^T^ isolated from peat bog soil in Baden, Germany was also pathogenic on onion. It is likely onion pathogenicity factors are conserved in *R. badensis.* Apart from confirming pathogenicity on onion (bulb and red scale) and fulfilling Koch’s postulates for all seven strains, we also confirmed if the isolated strains were indeed onion pathogens. The population dynamics of a representative onion-pathogenic *R. badensis* strain (20GA0316R) on red-onion scale were evaluated. The strain 20GA0316R could reach population levels comparable to an onion-pathogenic *P. ananatis* strain PNA 97-1R. These observations indicate that *R. badensis* strains isolated from onion are indeed pathogens of onion. It is not clear if this pathogen was introduced from a different geographical region into Georgia or expanded its host range to onion. Since limited genomic sequences are available in NCBI, efforts are underway to survey more commercial onion fields and storage facilities to get a large number of strains that can be further assessed using population genomic approaches to identify the potential source of introduction.

Foliar and bulb symptoms on onion caused by diverse bacterial pathogens rely on one or more virulence factors. To further explore the virulence attributes of *R. badensis,* the genomes were screened for typical virulence-associated genes. The *hrp-*T3SS is essential to the virulence of many well-characterized plant pathogenic bacteria. For example, hemibiotrophic bacterial pathogens such as *Pseudomonas* spp. and *Xanthomonas* spp. use *hrp-*T3SS to inject effector proteins directly into host cells to modulate plant host immunity and induce disease (Chang et al., 2014). Soft-rot necrotrophic pathogens such as *Dickeya* spp. and *Pectobacterium* spp. employ type II secretion system (T2SS) to deliver extracellular cell wall degrading enzymes that macerate host tissues (Chang et al., 2014). However, the complete clusters of plant-associated T3SS (Hrp types), T2SS, and T6SS genes were not found in *R. badensis* strains evaluated, which suggests that these commonly used virulence factors of plant-pathogenic bacteria are not potentially utilized by this bacterium to infect onion. Interestingly, two animal-associated T3SS clusters (Ssa-Esc and Inv-Mxi-Spa types) were found in *R. badensis* genomes, indicating potential interactions with animals, possibly arthropods, which is not surprising with bacteria belonging to Yersiniaceae family. The T1SS and T5SS were also found in all 10 *R. badensis* genomes. However, the roles of T1SS and T5SS in plant pathogenicity are unknown for this pathosystem in the absence of experimental evidence.

None of the seven *R. badensis* strains displayed foliar symptoms under greenhouse conditions, which suggests that these strains may prefer onion bulb or scale tissues rather than foliar tissues. The *R. badensis* strains also lacked the HiVir gene cluster, which has been reported as a main virulence factor responsible for foliar necrosis in *P. ananatis* (Asselin et al., 2018; Stice et al., 2020). It is possible that its absence in *R. badensis* may potentially indicate lack of bacterial ability to cause foliar lesions. This assertion needs to be further evaluated and proved experimentally. We acknowledge that the presence of HiVir cluster is not an indicative of onion-pathogenesis in most bacterial pathogens infecting onion other than in some specialized *P. ananatis* strains. Similar trend was also observed in case of onion-pathogenic *R. badensis* strains as this pathogenicity factor was absent in all pathogenic strains screened. These observations indicate that *R. badensis* may not utilize this pathogenicity factor for onion pathogenesis.

Interestingly, despite being isolated from symptomatic foliar tissue, the strain 20GA0206 and 21GA0654 were not able to induce foliar symptoms upon inoculation under our greenhouse conditions. It is likely that these strains might have been secondary foliar colonizers rather than a primary causal agent in the necrotized foliar tissue. It is possible that these strains were isolated accidently. Interestingly, *R. badensis* 20GA0206 and 21GA0654 were able to cause symptoms in onion bulbs and shared most of the phenotypic and genotypic attributes as that of other bulb-isolated *R. badensis* strains. Previous reports indicate that *Rouxiella* as a part of microbial communities in symptomatic onion bulbs (Yurgel et al., 2018). This shows the association of *Rouxiella* with onion bulb is not uncommon but their role as a bulb pathogen has never been observed nor previously characterized.

Secondary metabolites are associated with pathogenicity and virulence in plant-pathogenic bacteria. Among the 10 *R. badensis* genomes analyzed, the same nine gene clusters that potentially encode for the biosynthesis of secondary metabolites were identified in all strains, regardless of the isolation sources. Some of the secondary metabolites that these clusters potentially encode include those that were previously identified in other bacteria. For example, aryl polyenes are pigments produced by bacteria living in varied environment, and protect bacteria against photo-damage, oxidative stress, or reactive oxygen species (Schöner et al., 2016; He et al., 2020). Two secondary metabolite gene clusters detected in the *R. badensis* genomes were related to iron acquisition. One is a desferrioxamine E like siderophore product, and the other is a non-ribosomal peptide synthetase (NRPS) type product similar to turnerbactin. Turnerbactin is a triscatecholate siderophore that was first described in shipworm endosymbiont *Teredinibacter turnerae* (Han et al., 2013). In response to iron limitation, many bacteria and some fungi produce siderophores. Iron acquisition could potentially be crucial for its survival in soil and its ability to colonize onion host. Besides, lipopeptides were reported as virulence factors for several *Pseudomonas* species, including cichopeptins from *P. cichorii* causing lettuce midrib rot (Huang et al., 2015), corpeptin from *P. corrugate* causing tomato pith necrosis (Strano et al., 2015), tolassin from *P. tolaasii* causing bacterial blotch on cultivated mushrooms, and syringopeptin and syringomycin from *P. syringae* pv. *syringae* (Raaijmakers et al., 2006). It would be interesting to assess if these secondary metabolites play a role in onion pathogenicity in *R. badensis*. Future mutational analysis of genes and gene clusters related to secondary metabolites particularly lipopeptides will be evaluated for onion pathogenicity.

The presence of the *alt*-like cluster in onion-associated *R. badensis* strains likely facilitates colonization in onion bulbs. The fact that the *alt*-like cluster is enriched in strains isolated from symptomatic onion bulbs but not in strains from other isolation sources indicates potential competitive advantage of the former strains over the latter to colonize thiosulfinate-rich environment in onion bulbs. Similar observations were made in *Pantoea* strains where significant enrichment of the *alt* cluster was detected in strains that were recovered from symptomatic onion bulbs compared to strains isolated from other sources (Stice et al., 2021). A *P. fluorescens* strain from garlic exhibiting resistance to allicin, also uses gene clusters involved in redox-related functions (Borlinghaus et al., 2020). Strains from different genera use similar mechanisms to tolerate *Allium* thiosulfinates could imply that many bacterial pathogens associated with *Allium* species possess similar resistance mechanisms. Thus, we speculate that the *alt*-like cluster from *R. badensis* onion strains may also contribute to thiosulfinate-tolerance and play a role in onion bulb colonization. Further mutational analysis of genes in *alt* cluster will be evaluated for *R. badensis*’ ability to colonize onion bulbs and tolerate allium thiosulfinates.

In conclusion, in this study, we report *R. badensis* as a plant pathogen for the first time, causing onion bulb rot. The seven onion *R. badensis* strains were pathogenic on onion bulb tissues, but not on foliar tissues. Genome analysis and secondary metabolite prediction provide new insights into *Rouxiella*-host interactions that warrant further characterization. The contributions of the *alt-*like gene cluster and predicted secondary metabolite gene clusters such as lipopeptide remain to be determined. Future studies will involve investigating the role of the *alt*-like cluster and lipopeptides in onion pathogenicity for the *R. badensis* strains associated with onion.

## Data availability statement

The datasets presented in this study can be found in online repositories. The names of the repository/repositories and accession number(s) can be found below: https://www.ncbi.nlm.nih.gov/bioproject/?term=PRJNA776088, BioProject PRJNA776088.

## Author contributions

MZ and BD conceived the project. CT provided samples. MZ conducted the experiments and analysis. RG and BK critically reviewed the manuscript. BK and BD planned the project and secured extramural funds. All authors contributed to the manuscript and approved the submitted version.

## Funding

This work was supported by the Specialty Crops Research Initiative Award 2019–51181-30013 from the USDA National Institute of Food and Agriculture. Any opinions, findings, conclusions, or recommendations expressed in this publication are those of the author(s) and do not necessarily reflect the view of the U.S. Department of Agriculture. The University of Georgia is an equal opportunity provider and employer. This study was supported in part by resources and technical expertise from the Georgia Advanced Computing Resource Center, a partnership between the University of Georgia Office of the Vice President for Research and Office of the Vice President for Information Technology.

## Conflict of interest

The authors declare that the research was conducted in the absence of any commercial or financial relationships that could be construed as a potential conflict of interest.

## Publisher’s note

All claims expressed in this article are solely those of the authors and do not necessarily represent those of their affiliated organizations, or those of the publisher, the editors and the reviewers. Any product that may be evaluated in this article, or claim that may be made by its manufacturer, is not guaranteed or endorsed by the publisher.

## Supplementary material

The Supplementary material for this article can be found online at: https://www.frontiersin.org/articles/10.3389/fmicb.2022.1054813/full#supplementary-material

## Footnotes

^1^ https://microbializer.tau.ac.il/index.html

^2^https://effectors.csb.univie.ac.at/)
