# Supplementary material for: Rouxiella badensis, a new bacterial pathogen of onion causing bulb rot
Source: Front Microbiol. 2022 Nov 30;13:1054813. doi: 10.3389/fmicb.2022.1054813 (PMC9749400; doi:10.3389/fmicb.2022.1054813)
Supplement: Supplementary file 1 [file Data_Sheet_1.docx]

Supplementary Material


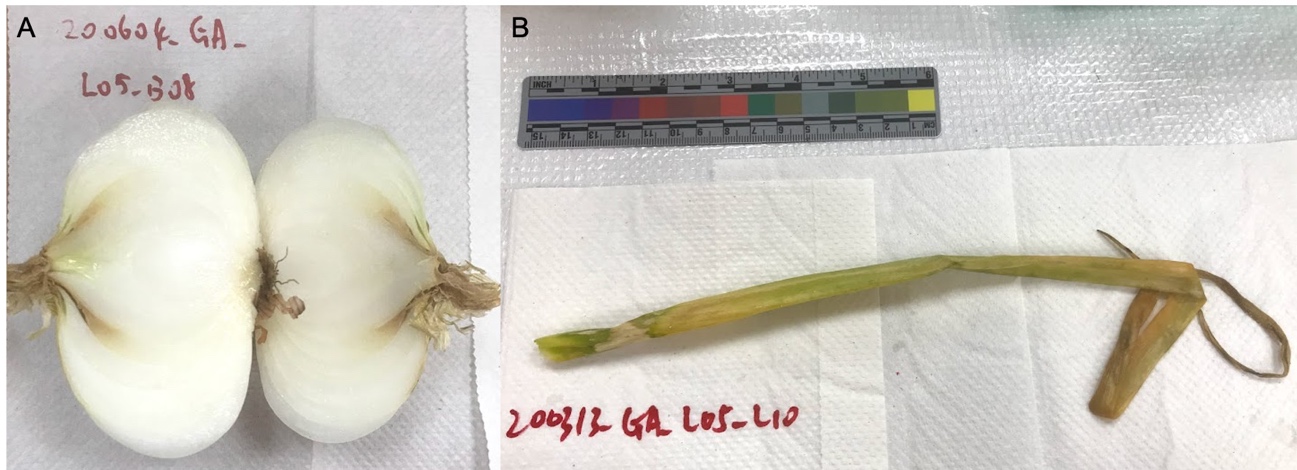


**Supplementary Figure 1.** Representative symptom of an original isolation. Symptoms on an onion bulb included discoloration of internal scales.
